# Artificial Intelligence-Assisted Prediction of Midpalatal Suture Maturation from Cervical Vertebral Maturation Assessed on Lateral Cephalometric Radiographs: Development of a Combined Diagnostic Model

**DOI:** 10.3390/diagnostics16213568

**Published:** 2026-10-29

**Authors:** Saliha Tutar, Esra Bolat Gumus, Turgut Felek, Ali Rasat

**Affiliations:** 1 Department of Orthodontics, Faculty of Dentistry, Akdeniz University, 07058 Antalya, Türkiye; dtesrabolat@gmail.com; 2 Department of Information Technologies, Akdeniz University, 07058 Antalya, Türkiye; turgutfelek@akdeniz.edu.tr; 3 Department of Maxillofacial Radiology, Faculty of Dentistry, Burdur Mehmet Akif Ersoy University, 15100 Burdur, Türkiye; alicanras07@hotmail.com

**Keywords:** midpalatal suture maturation, cervical vertebral maturation, artificial intelligence, deep learning, YOLOv11

## Abstract

**Background/Objectives**: This study evaluated the association between cervical vertebral maturation (CVM) and midpalatal suture (MPS) maturation and developed an artificial intelligence (AI)-assisted model for predicting MPS stage from lateral cephalometric radiographs and clinical variables. **Methods**: CVM and cone-beam computed tomography (CBCT)-defined MPS stages were assessed in 250 individuals. Their association was examined using Spearman correlation and multivariable ordinal logistic regression incorporating CVM stage, chronological age, and sex. A separate dataset of 1448 cephalograms was used to develop and evaluate a YOLOv11 model for automated CVM classification. The combined-model cohort comprised 325 patients, including the initial 250 and 75 additional patients, divided into training (*n* = 260) and held-out internal test (*n* = 65) subsets. The combined model was trained using reference CVM stage, chronological age, and sex and evaluated on the held-out test set using AI-predicted CVM stage in place of reference CVM. **Results**: CVM and MPS stages were strongly correlated (r = 0.749, *p* < 0.001). CVM stage and age were significantly associated with MPS stage, whereas sex was not (Nagelkerke R^2^ = 0.716). YOLOv11 correctly detected and classified 277/344 reference regions (80.5%). On the held-out test set, the combined model achieved 69.2% five-class accuracy (45/65; 95% CI: 57.2–79.1%) and 92.3% binary accuracy for A–C versus D–E. Repeated nested cross-validation across 325 patients yielded 58.3% mean accuracy with reference CVM, compared with 58.8% for reference CVM alone. Substituting AI-CVM only during evaluation in the 65-patient subset yielded 55.7% mean accuracy. **Conclusions**: The proposed AI-assisted model remains exploratory, with automated-input evaluation limited to 65 patients. The added predictive value of age and sex beyond CVM staging alone remains unproven. Its performance does not establish the ability to predict expansion success or justify replacing clinically indicated CBCT. External validation is required before clinical implementation.

## 1. Introduction

Accurate assessment of midpalatal suture maturation is essential for determining the appropriate timing and expected skeletal response to rapid maxillary expansion (RME). Patients treated before the peak of puberty exhibit significant, more effective long-term changes at the skeletal level in both maxillary and circummaxillary structures. In treatments performed after the adolescent growth spurt, maxillary adaptations for expansion treatment shift from the skeletal to the dentoalveolar level [1]. This situation can lead to complications such as alveolar bone loss and tooth resorption, gingival recession, and relapse, along with unwanted periodontal stress [2,3,4,5]. Therefore, accurately determining the MPS stage to optimize treatment timing and avoid invasive procedures is one of the fundamental goals of modern orthodontics.

The response to rapid maxillary expansion can be unpredictable in late adolescent and young adult patients. Individual variation in midpalatal suture maturation limits the use of chronological age alone to infer sutural stage, highlighting the need for individualized assessment [6,7,8]. To facilitate this assessment, Angelieri et al. [9] introduced a CBCT-based classification distinguishing five maturation stages (A–E) according to sutural morphology and radiographic evidence of fusion. However, CBCT is not a practical or ethical option for every patient, as it involves a higher radiation dose and is not always included in routine orthodontic diagnostic protocols. This has led clinicians to make indirect inferences using less invasive, more commonly used radiographs.

At this point, a commonly used method for assessing an individual’s skeletal maturation is cervical vertebra maturation analysis performed on routinely acquired cephalometric radiographs. Baccetti et al. [10] classified the cervical stage (CS) of an individual according to their pubertal growth spurt by examining the morphology of the second, third, and fourth cervical vertebrae. Previous studies have reported significant associations between MPS maturation and skeletal maturity assessed using cervical vertebral maturation stages or hand–wrist radiographs [11,12,13,14]. However, Ferrillo et al. [7] found that CVM staging was not a useful predictor of MPS maturation in their cohort, indicating that findings are not consistent across studies. These differing findings highlight the need to distinguish population-level associations from predictive accuracy at the individual patient level. Taken together, these findings suggest that skeletal maturation may provide indirect information regarding MPS stage and may help inform the overall timing of orthodontic treatment. However, manual CVM analysis is an experience-based, subjective process that can lead to inconsistencies among clinicians performing the assessment and even between assessments by the same clinician at different times [15]. This subjectivity makes standardizing diagnosis difficult and time-consuming.

In recent years, artificial intelligence has been increasingly explored across clinical processes in medicine and dentistry, including diagnosis, treatment planning, and patient monitoring. In particular, machine learning and deep learning algorithms can perform automated image diagnosis and classification with high accuracy [16]. Applying this technology to orthodontic radiography analysis automates diagnostic processes, making diagnosis faster, more objective, and more consistent. In the field of skeletal maturation assessment, convolutional neural networks have been investigated for automated CVM classification from lateral cephalometric radiographs [17,18,19]. Detection-based approaches, including YOLO-based systems, have also been developed for automated CVM assessment [20,21], providing a methodological basis for incorporating automated skeletal staging into subsequent prediction models. Recent studies have investigated automated MPS assessment using CBCT-based deep learning, multimodal models incorporating imaging and clinical variables, and direct prediction of CBCT-defined MPS maturity from lateral cephalograms [22,23,24]. Within this context, the present study evaluates a two-stage approach that combines automated CVM classification from lateral cephalograms with chronological age and sex to predict the five MPS maturation stages.

In this context, this study is based on two main hypotheses: (1) CVM stages obtained from cephalometric radiographs, together with data such as chronological age and sex, can form a statistically significant model for predicting MPS stage. (2) A modern deep learning algorithm, such as YOLOv11, can be used for automated detection and classification of CVM stages from lateral cephalometric radiographs.

Therefore, the aims of this study were (1) to investigate the relationship between CVM and MPS maturation, (2) to develop and validate a YOLOv11-based model for automated CVM classification, and (3) to construct a combined diagnostic model for predicting MPS maturation using automated CVM stage, age, and sex.

## 2. Materials and Methods

Our study was approved by Akdeniz University Medical Scientific Research Ethics Committee on 30 May 2024 with decision number TBAEK-365. This study is a single-center, retrospective, and cross-sectional study evaluating cephalometric radiographs and CBCT records of individuals aged 8–25 years who sought orthodontic treatment between 2019 and 2025. The inclusion of records dated after the ethics approval reflects the subsequent addition of 75 patients to the combined-model cohort. These patients were selected using the same eligibility criteria from records added to the institutional archive between October 2024 and May 2025. All imaging examinations were performed for routine clinical indications, and the existing records were subsequently reviewed for research purposes. No additional imaging was performed specifically for this study. The study was conducted in three phases: the first analyzed the relationship between CVM and MPS, the second performed automated CVM classification, and the third developed and internally validated a combined model incorporating clinical variables. A general overview summarizing the purpose, sample size, and content of the datasets used in the study is presented in Table 1.

The following criteria were considered in selecting individuals for the study:

- Absence of any systemic or metabolic disorders and syndromes (cleft lip and palate, cleidocranial dysplasia, etc.),
- No prior maxillary expansion or orthognathic surgery treatment,
- No more than three months between the acquisition dates of cephalometric radiography and CBCT images,
- Availability of images of evaluable quality.

### 2.1. Determination of Cervical Vertebral Maturation Stage

Cephalometric radiographs of the 250 individuals included in the study were obtained digitally using a Planmeca 2002 CC Proline Pan/Ceph X-Ray (Planmeca Oy, Helsinki, Finland). The distance between the radiation source and the film was standardized as 160 cm, and the distance between the midsagittal plane and the film as 16 cm. The raw images selected for the study were exported in Joint Photographic Experts Group (JPEG) format using Romexis software (v3.5, Planmeca Oy, 00880 Helsinki, Finland). Each image was numbered, recording the sex, birth dates, imaging dates, and chronological ages of the individuals. The patients’ ages were calculated by subtracting their birth dates from the imaging dates.

**Figure 1:**
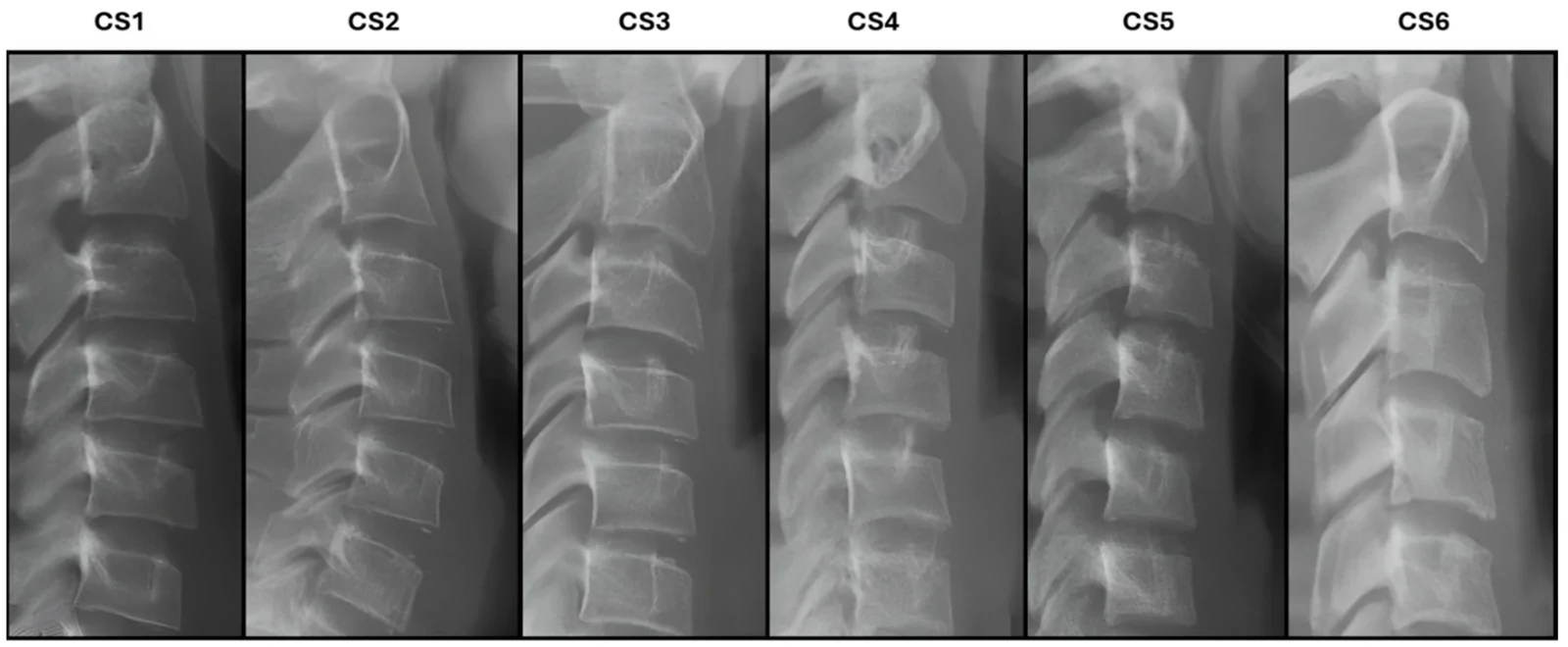
Classification of cervical vertebral maturation stages.

The six-stage classification by Baccetti et al. [10] was used to classify cervical vertebral maturation. The classification was performed independently by two examiners (S.T., A.R.), and both examiners were blinded to the corresponding MPS stage and chronological age during CVM assessment. Disagreements were resolved by jointly reviewing the images and reaching consensus. Inter-examiner agreement was 0.898 (95% CI: 0.862–0.928) using linearly weighted kappa and 0.965 (95% CI: 0.950–0.976) using quadratically weighted kappa. Sample images of the cervical vertebral maturation stages determined in our study are presented in Figure 1.

### 2.2. Determination of Midpalatal Suture Maturation Stage

CBCT images of 250 individuals included in the study were obtained using a Veraview X800 device (J. Morita Mfg. Corp., Kyoto, Japan) according to the manufacturer’s instructions (FOV: 15 × 14; 4.8 mA; 99 kVP and 35.8 s). Scans were analyzed using i-Dixel software (version 2.3.6.1; J. Morita Mfg. Corp., Kyoto, Japan). Image acquisition was performed with a single 360° rotation of the device around the patient. All acquired images were saved in DICOM (Digital Imaging and Communications in Medicine) format. The sex, birth dates, imaging dates, and chronological ages of the individuals were recorded, and each image was numbered. The patients’ ages were calculated by subtracting their birth dates from their imaging dates.

**Figure 2:**
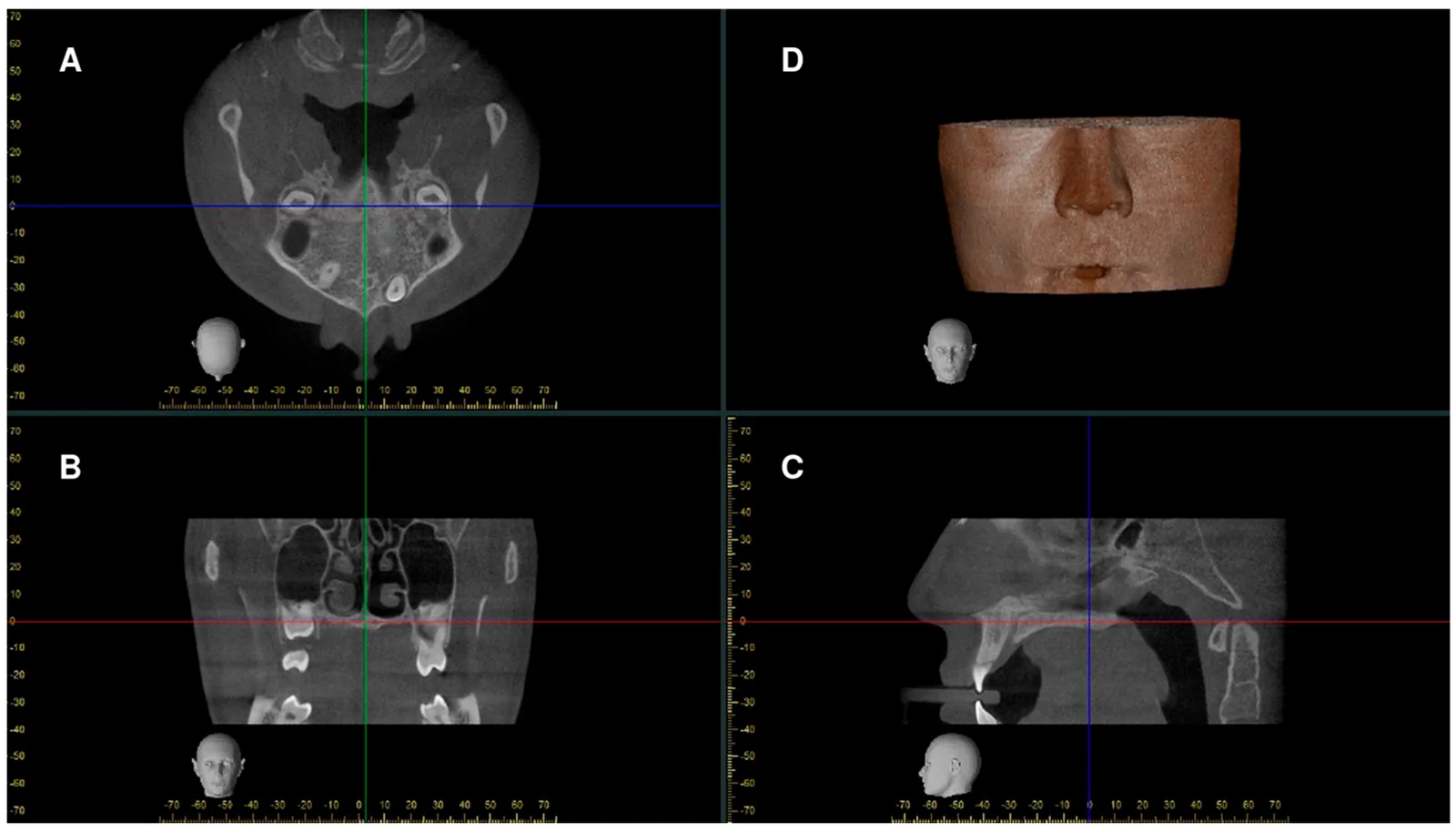
Standardization of head position for the assessment of midpalatal suture maturation: (**A**) axial view; (**B**) coronal view; (**C**) sagittal view; (**D**) three-dimensional reconstruction. The red, blue, and green reference lines indicate the axial, coronal, and sagittal planes, respectively.

The MPS stage was determined from the CBCT images using the classification system defined by Angelieri [9]. To allow for consistent assessment of the midpalatal suture, head positioning was standardized. Natural head positioning was verified or corrected in all three planes of space. A position indicator was placed in the patient’s mid-sagittal plane in both coronal and axial views (Figure 2A,B). In the sagittal view, the patient’s head was positioned so that the anteroposterior long axis of the palate was horizontal (Figure 2C).

**Figure 3:**
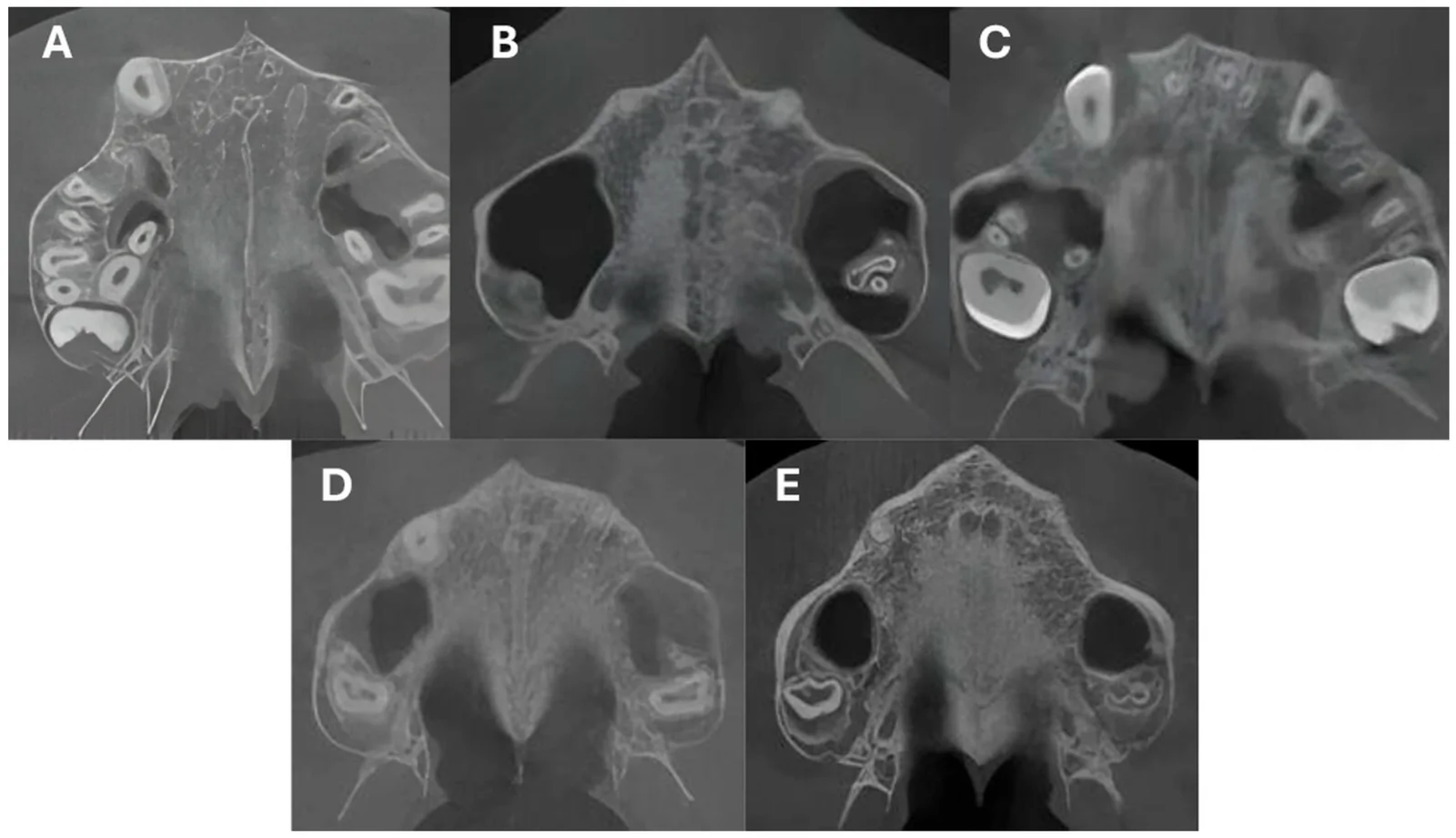
Classification of midpalatal suture maturation stages. (**A**) Stage A; (**B**) Stage B; (**C**) Stage C; (**D**) Stage D; (**E**) Stage E.

Classification was performed independently by two examiners (S.T., A.R.), and both examiners were blinded to the corresponding CVM stage and chronological age during MPS assessment. Disagreements were resolved by jointly reviewing the images and reaching consensus. Inter-examiner agreement was 0.737 (95% CI: 0.678–0.789) using linearly weighted kappa and 0.872 (95% CI: 0.835–0.901) using quadratically weighted kappa. Sample images of the midpalatal suture maturation stages determined in our study are presented in Figure 3.

### 2.3. Artificial Intelligence Dataset Preparation

**Figure 4:**
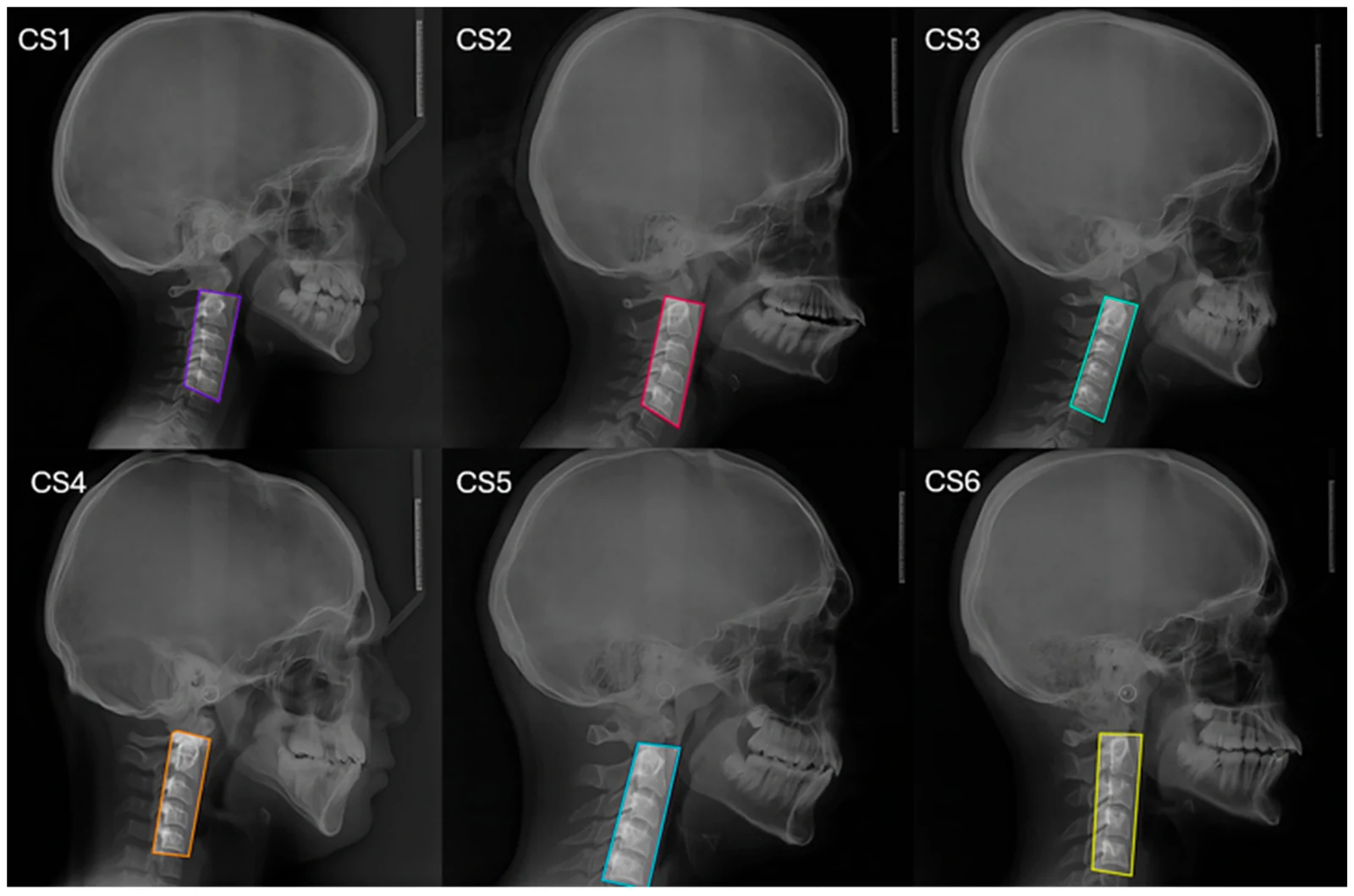
Labeling the dataset via Roboflow. The colored bounding boxes delineate the annotated cervical vertebral regions, with different colors used to distinguish the CVM stages (CS1–CS6).

During the development phase of the artificial intelligence model, 1448 digital cephalometric radiographs of patients aged 8–25 years were obtained from the Department of Oral, Dental, and Maxillofacial Radiology archive at the Faculty of Dentistry, Akdeniz University. Chronological age was calculated by subtracting each individual’s birth date from the radiograph recording date. The images were examined and labeled. Cervical vertebral maturation stages were classified into six stages according to the classification by Baccetti et al. [10]. The classification process was repeated after two weeks for 300 randomly selected patients, and intra-examiner agreement for CVM staging was assessed using linearly weighted kappa (wk = 0.89). All labels were generated by an orthodontist experienced in CVM assessment (S.T.) via Roboflow (https://roboflow.com/) and reviewed by a dentomaxillofacial radiologist (A.R.). Disagreements were resolved by consensus. Classes representing the targeted samples were defined, and a different color was assigned to each to facilitate class recognition (Figure 4).

**Figure 5:**
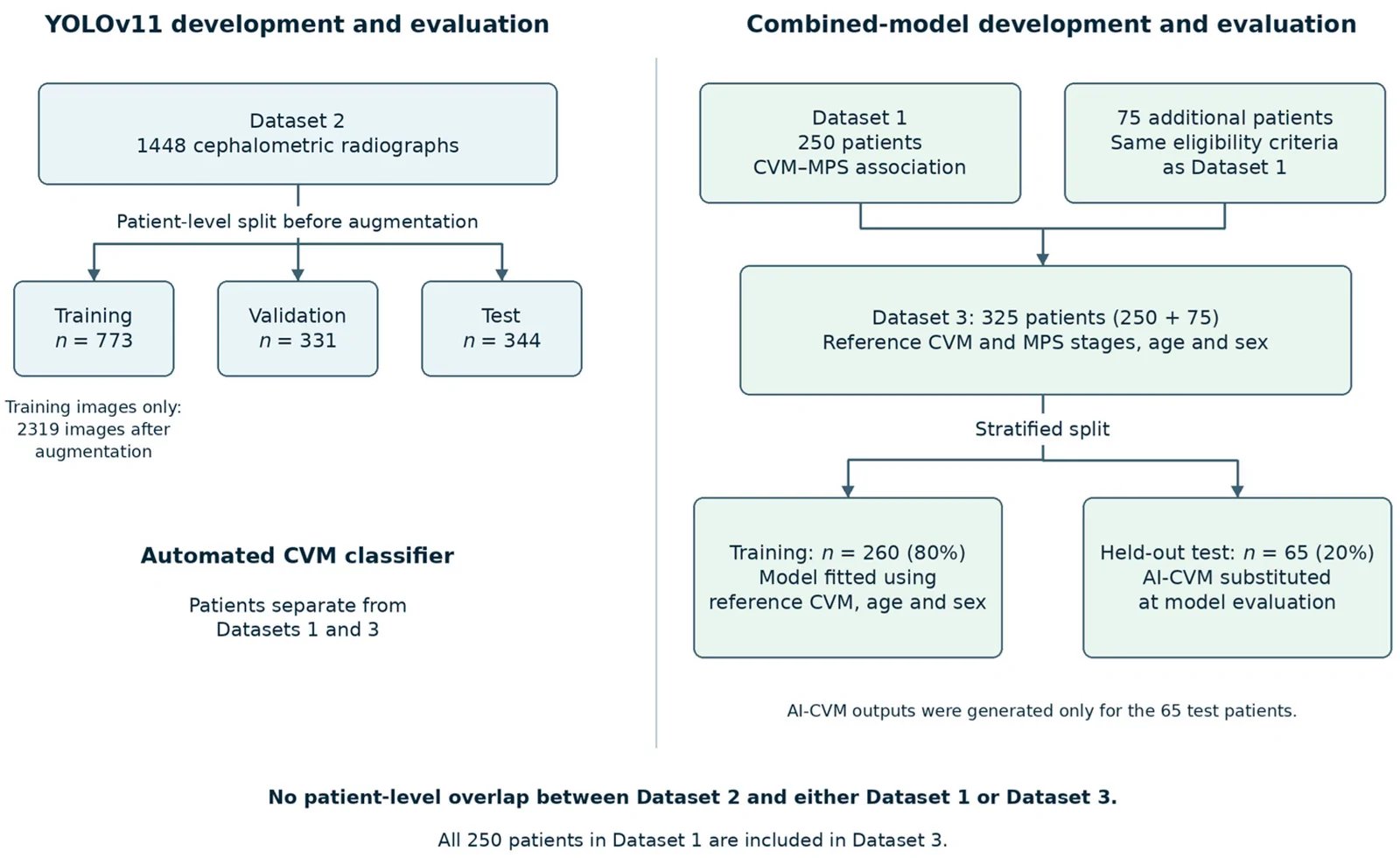
Relationships and patient-level overlap among the three datasets. Dataset 3 comprised all 250 patients from Dataset 1 and 75 additional eligible patients. Dataset 2 had no patient-level overlap with either Dataset 1 or Dataset 3. Training, validation, and test allocations are shown for the respective models.

In our dataset, the number of cephalometric radiographs for cervical stages CS1–CS6 was 245, 228, 235, 240, 251, and 249, respectively. The 250 patients in Dataset 1 were not included in any of the training, validation, or test subsets of the 1448-image dataset used to develop and evaluate the YOLOv11 model. Access to the dataset was provided via a Roboflow API key, and the dataset was integrated into the Google Colab (runtime version 2026.04; Python 3.12.13; PyTorch 2.10.0) runtime environment using the Roboflow Python SDK (version 1.3.7). After the datasets were created, training, validation, and test datasets were generated (Figure 5).

During data augmentation, three outputs were generated for each training sample, yielding 2319 images. To prevent overfitting the training dataset, combinations of various data augmentation techniques were used, including random noise (0.1%), rotation (±15°), zoom (±15%), and brightness (±15%). The dataset was split at the patient level before augmentation, so that all images of a given patient were assigned to the same subset, and augmentation was applied only to the training images.

### 2.4. Model Architecture and Training Process

For automatic detection and classification of CVM stages, the YOLOv11 object detection algorithm (Ultralytics, version 8.3.40) was selected. Model training was performed on Google Colab using an NVIDIA T4 GPU (NVIDIA Corporation, Santa Clara, CA, USA). Images were pre-processed and made suitable for network input. Batch size: 32, learning rate: 0.001, number of epochs: 60 parameters were used in the training process. The trained model’s outputs were visualized using the supervision library (version 0.28.0), and predicted class labels and bounding boxes were obtained for each CVM. Additionally, the Roboflow Inference (version 1.2.6) service was used to obtain CVM stage predictions from cephalometric radiographs.

The correct detection-and-stage classification rate was defined as the number of reference CVM regions that were detected and assigned the correct stage divided by the total number of reference regions. Missed detections and incorrect stage assignments were counted as errors. Class-specific precision, recall, and F1-score were calculated as TP/(TP + FP), TP/(TP + FN), and 2TP/(2TP + FP + FN), respectively. Stage misclassifications contributed a false positive to the predicted class and a false negative to the reference class. Unmatched predictions contributed false positives, and missed reference regions contributed false negatives.

### 2.5. Development of the Combined Diagnostic Model

The combined model used CVM stage, chronological age, and sex as predictors of MPS stage. The model was fitted using the reference (consensus manual) CVM stages of the training patients; at the prediction step, the CVM input was the stage assigned by YOLOv11, so that the evaluated pipeline required no manual CVM staging. The cohort comprised the 250 patients in Dataset 1 and 75 additional patients selected from eligible records added to the institutional archive between completion of the initial cohort selection and availability of the YOLOv11 training results. The same eligibility criteria were applied to both groups. None of these 325 patients was included in the training, validation, or test subsets of the YOLOv11 dataset (Dataset 2). The combined-model cohort was divided into training (*n* = 260, 80%) and held-out test (*n* = 65, 20%) subsets using a stratified split. The combined model was implemented using an XGBoost classifier within a pipeline, and the fitted model was saved in joblib (version 1.5.3) format.

For repeated nested cross-validation, the hyperparameter search comprised eight combinations of n_estimators (200 or 600), max_depth (2 or 4), and learning_rate (0.05 or 0.08). Fixed settings were objective = ‘multi:softprob’, num_class = 5, tree_method = ‘hist’, eval_metric = ‘mlogloss’, subsample = 0.8, colsample_bytree = 0.8, min_child_weight = 1, gamma = 0, reg_alpha = 0, and reg_lambda = 1. No class weighting, SMOTE, data augmentation, or early stopping was used for the combined model. Hyperparameters were selected separately within each outer training partition and were not constrained to be identical across the 25 outer-fold evaluations.

The model evaluated on the held-out test set was fitted with n_estimators = 600, max_depth = 4, learning_rate = 0.08, subsample = 0.8, colsample_bytree = 1.0 and random_state = 42. These hyperparameters were selected using five-fold stratified cross-validated grid search, with macro-F1 as the selection metric, within the training data of a preliminary analysis. None of the 65 patients subsequently assigned to the final held-out test set participated in this hyperparameter-selection procedure, and their outcomes did not influence the selected values. The selected hyperparameters were then fixed for training the final held-out model. YOLOv11 inference was performed only for the 65 held-out test patients. Automated CVM outputs were not required for model fitting because the model was trained using reference CVM stages. Automated-input evaluation was conducted in the 65 held-out patients, none of whom participated in YOLOv11 development or training of the final held-out combined model. If more than one CVM bounding box was returned, the prediction with the highest confidence was used; predictions were not filtered by confidence, and a cephalogram without detection was to be counted as an incorrect MPS prediction. In a secondary cross-validation analysis, the models fitted within each outer training partition were additionally evaluated in members of this 65-patient subset only when those patients belonged to the corresponding outer validation fold, replacing reference CVM with the YOLOv11-predicted stage without refitting.

To comprehensively evaluate the predictive contribution of the artificial intelligence pipeline, several baseline and reference configurations were evaluated and compared on the held-out internal test set (*n* = 65): (1) chronological age alone, (2) automated CVM stage alone (AI-CVM), (3) manual CVM stage alone, (4) the same combined model evaluated using reference CVM stage, chronological age, and sex, and (5) a classic clinical heuristic rule defining CS1–CS3 as open sutures (stages A–C) and CS4–CS6 as fused sutures (stages D–E). Additional internal validation used repeated nested cross-validation with patient-level grouping. The outer loop comprised five-fold StratifiedGroupKFold cross-validation repeated with five random seeds (42, 123, 2026, 2027, and 2028), yielding 25 outer-fold evaluations. Within each outer training partition, hyperparameters were selected using three-fold StratifiedGroupKFold cross-validation, with macro-F1 as the selection metric. Patient groups were kept separate between training and validation partitions at both levels. The classifier random state was set to the repeat seed plus the fold index. Analyses were implemented in Python 3.13.15 using XGBoost 3.4.1 and scikit-learn 1.6.1.

### 2.6. Statistical Analysis

An a priori sample size calculation for the CVM–MPS correlation analysis was performed using G*Power (version 3.1.9.7). A minimum of 138 participants was required to detect a correlation of r = 0.30 with a two-sided significance level of 0.05 and 95% power. This calculation applied to the correlation analysis and did not determine the sample size required for evaluating the combined model’s classification performance. The combined-model cohort comprised 325 eligible patients, of whom 65 were allocated to the held-out test set. At the same time, the relationship between the CVM and MPS stages, as determined by the combined diagnostic model and the manually determined stages, was analyzed using the weighted kappa statistic.

Spearman correlations were evaluated using 99,999 permutations, with 95% confidence intervals obtained from 50,000 bootstrap resamples. Sex-specific correlations were compared using separate within-sex bootstrap resampling, with an approximate Fisher z test as a sensitivity analysis. Ordinal regression included CVM stage, chronological age, and sex; proportional odds were assessed using a score test. Sensitivity analyses evaluated alternative CVM codings, and profile-likelihood confidence intervals were calculated for early-stage coefficients.

For a secondary binary evaluation, reference and predicted MPS stages were grouped as A–C versus D–E, with A–C designated as the positive category. Binary accuracy, sensitivity, specificity, positive predictive value, and negative predictive value were calculated. Within-one-stage agreement was defined as the proportion of predictions differing from the reference stage by no more than one ordinal category. Wilson 95% confidence intervals were calculated for five-class accuracy, binary performance proportions, and within-one-stage agreement in the held-out test set.

For repeated cross-validation, performance metrics were calculated from the complete out-of-fold predictions within each repeat and then averaged across the five repeats. The 95% confidence intervals were estimated using 2000 patient-level cluster bootstrap resamples, retaining all records and repeated predictions belonging to each sampled patient. These intervals were conditional on the fitted models and fixed out-of-fold predictions; models were not refitted during bootstrap resampling.

## 3. Results

Descriptive statistics obtained from individuals within the scope of the study are presented in Table 2.

### 3.1. Results Regarding the Relationship Between CVM and MPS

The distribution of MPS stages observed at each CVM stage level, along with the statistical analyses related to this distribution, is presented in Table 3.

Table 3 shows that as the CVM stage progresses, the MPS stage shifts steadily to more advanced stages. A–B are dominant in CS1–CS2 stages, while D–E stages become more prominent in CS5–CS6 stages. The greatest overlap of MPS categories occurred at CS4 and CS5.

The distribution of CVM stage levels and MPS stages across sex groups, and the statistical relationships between these variables, are presented in Table 4.

The Spearman correlation between CVM and MPS stages was 0.851 in males (95% bootstrap CI, 0.769–0.907) and 0.680 in females (95% bootstrap CI, 0.571–0.772); both permutation *p*-values were <0.001. The unadjusted male-minus-female correlation difference was 0.171 (95% bootstrap CI, 0.048–0.294). An approximate Fisher z sensitivity analysis yielded z = 3.203 and *p* = 0.0014. CS5–CS6 accounted for 123/161 females (76.4%) and 55/89 males (61.8%), indicating different CVM-stage distributions between groups.

The categorical CVM model yielded a Nagelkerke R^2^ of 0.716. The proportional odds score test showed no statistically significant evidence of violation (χ^2^ = 31.570; df = 21; *p* = 0.065). Given the sparse early-stage CVM–MPS combinations and concerns about the stability of the CS1–CS3 coefficient estimates, sensitivity analyses were conducted using CVM as a single ordinal score (1–6) and combining CS1–CS3 into one category. Both alternatives showed poorer model fit, and the single-score model also showed evidence against proportional odds. The categorical model was therefore retained; however, these findings do not eliminate the uncertainty associated with sparse early-stage data. Predictor coefficients are presented in Table 5. Model comparisons, profile-likelihood confidence intervals, and model thresholds are provided in Supplementary Tables S1–S3.

The multivariable ordinal logistic regression results showed that CVM stage and chronological age were significantly associated with MPS stage, whereas sex was not. Compared with CS6, all lower CVM stages were associated with lower cumulative odds of being classified in a higher MPS category. For example, participants at CS5 had approximately 63% lower cumulative odds of being in a higher MPS category rather than at or below a given threshold (adjusted OR = 0.370; 95% CI: 0.186–0.737; *p* = 0.005). Chronological age was independently associated with MPS stage, with each additional year associated with approximately 31% higher cumulative odds of being in a more advanced category (adjusted OR = 1.313; 95% CI: 1.155–1.491; *p* < 0.001). Sex was not significantly associated with MPS stage (male vs. female: β = −0.396; adjusted OR = 0.673; 95% CI: 0.364–1.244; *p* = 0.206). The positive age association and nonsignificant sex association were also observed in both sensitivity models.

### 3.2. Results Regarding Automatic CVM Classification with Artificial Intelligence Model

**Figure 6:**
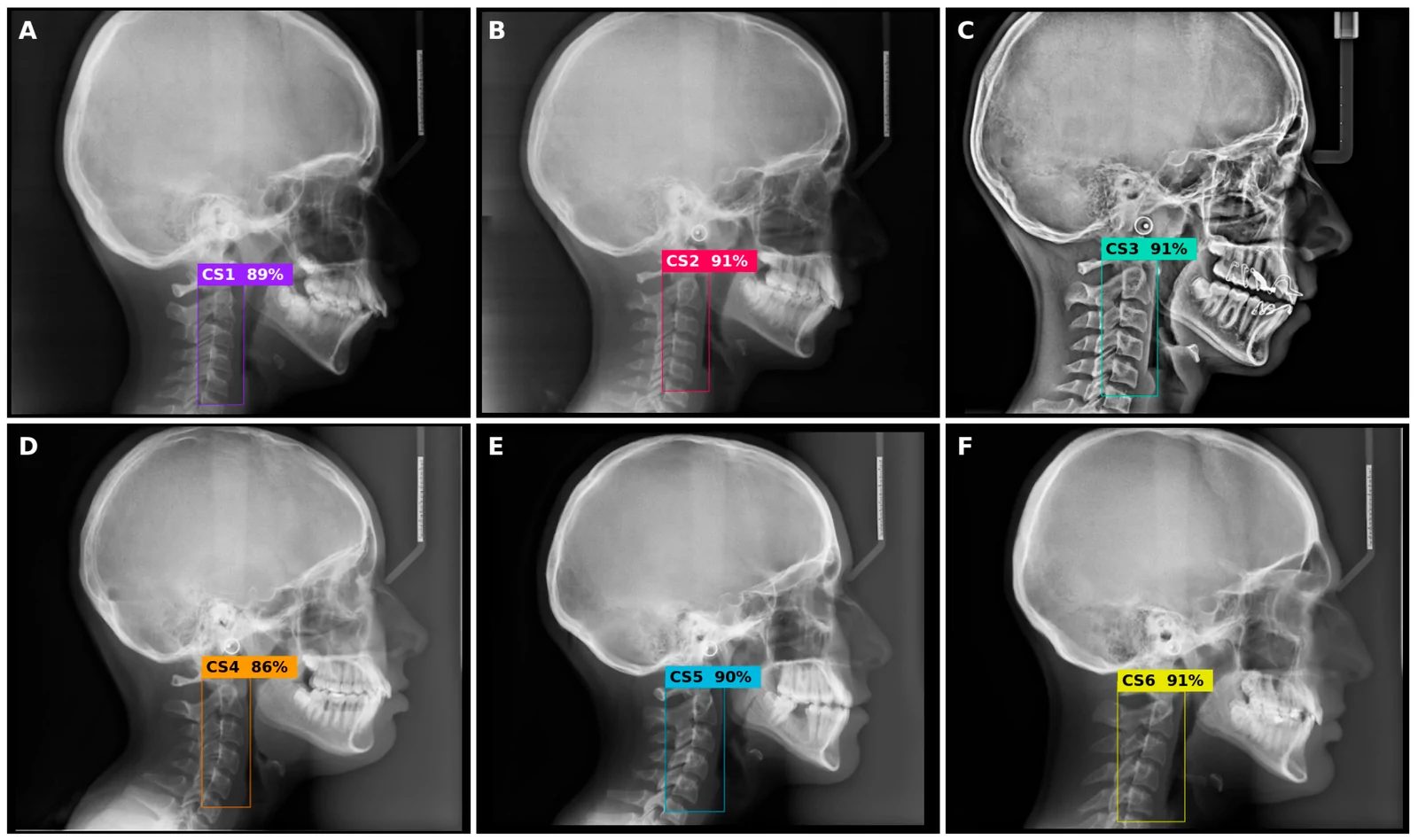
Classification outputs of the CSs of the YOLOv11 model. (**A**) CS1, 89%; (**B**) CS2, 91%; (**C**) CS3, 91%; (**D**) CS4, 86%; (**E**) CS5, 90%; (**F**) CS6, 91%. The colored bounding boxes indicate the detected cervical vertebral regions, with different colors distinguishing the predicted CVM stages. Percentages indicate model confidence scores for the displayed predictions. Figure 7 shows how the YOLOv11 model’s loss functions changed during training and validation (epochs). Loss is a measure of how much the model’s predictions deviate from the actual values. The goal is to minimize this loss value as much as possible.

Representative YOLOv11 outputs showing localization of the cervical vertebral region, predicted CVM stages (CS1–CS6), and confidence scores are presented in Figure 6.

**Figure 7:**
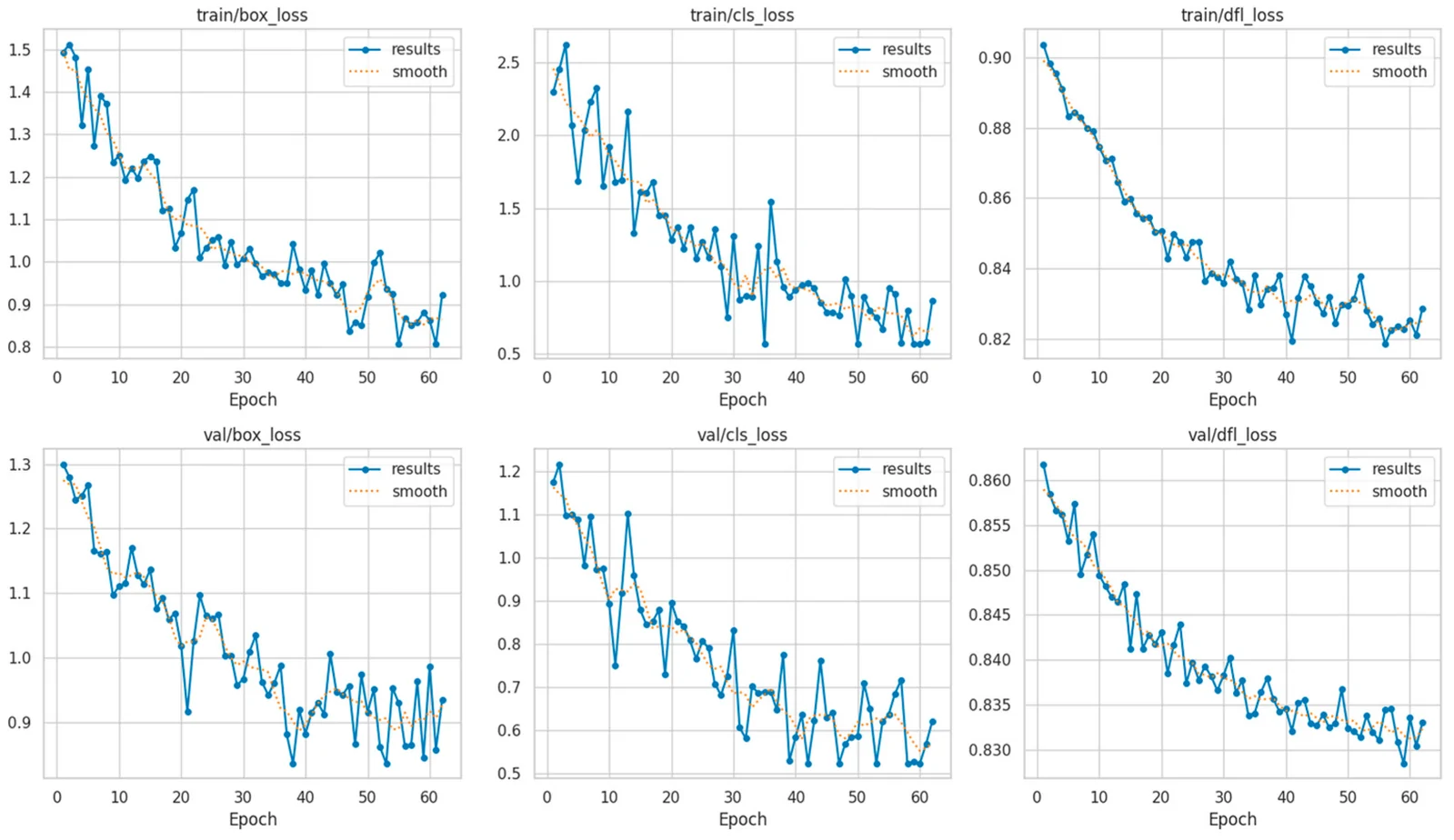
Loss functions of the YOLOv11 model during training and validation.

Training and validation losses decreased throughout training and remained closely aligned.

**Figure 8:**
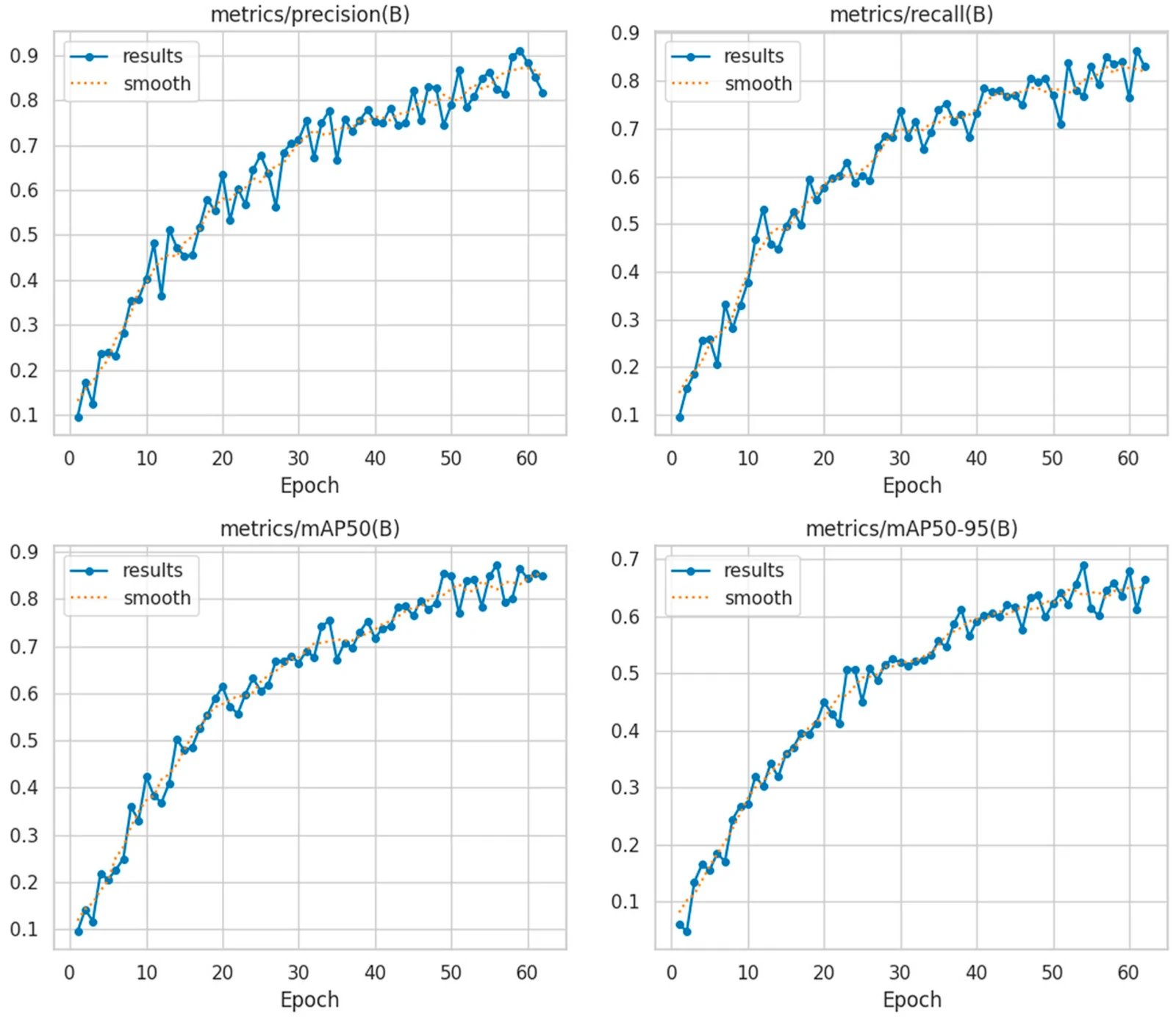
Performance metrics for the YOLOv11 model’s training and validation.

Figure 8 shows how the performance metrics of the YOLOv11 model changed during training and validation over the course of the training period (epochs). Unlike loss functions, the aim with these metrics is to bring the values as close to 1 as possible.

Precision, recall, mAP@0.50, and mAP@0.50–0.95 increased progressively during training and approached a plateau by epoch 60, with final test-set values of 85.0%, 80.5%, 87.35%, and 60.12%, respectively.

**Figure 9:**
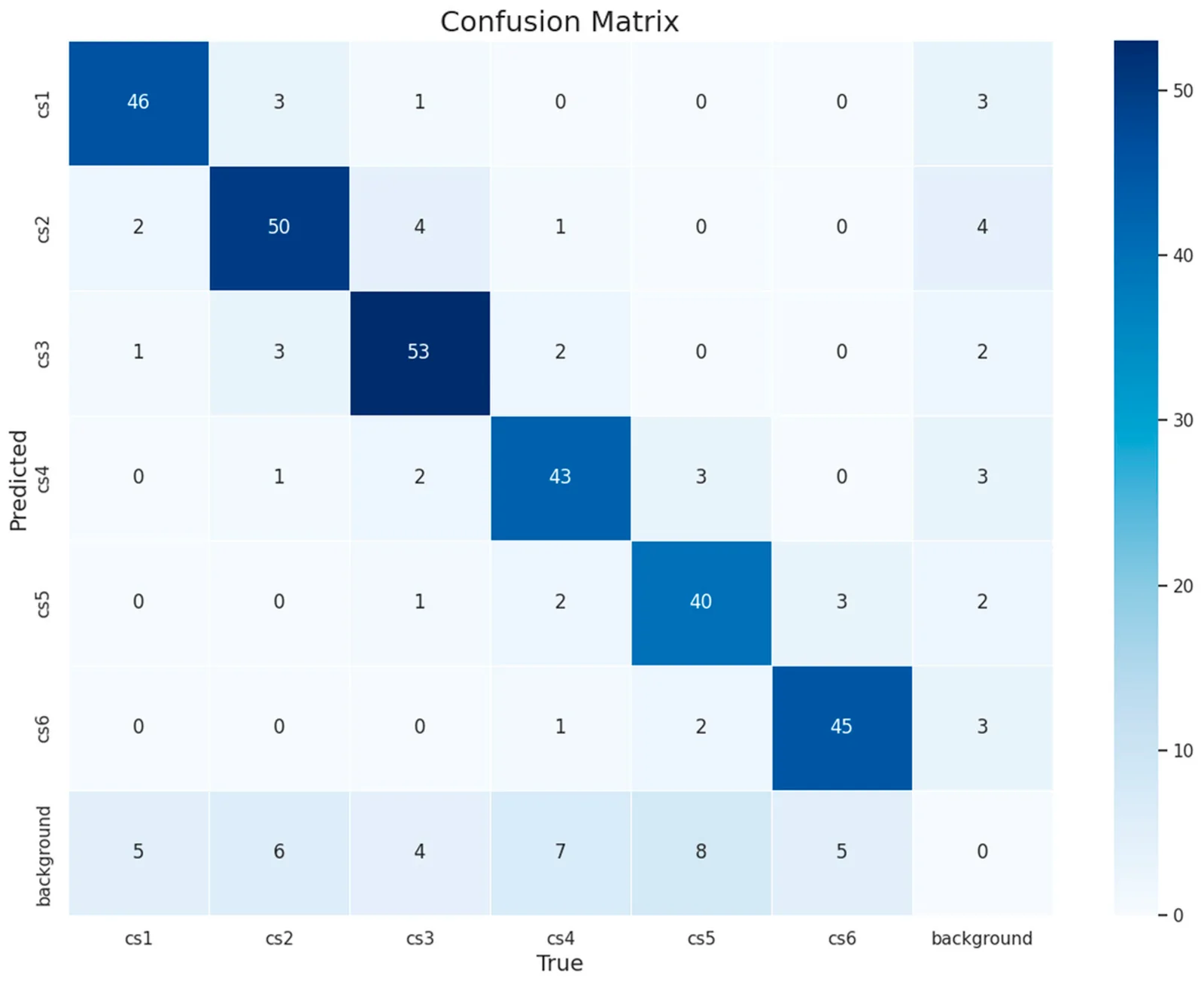
Confusion matrix for YOLOv11 evaluated on the 344-image test set of Dataset 2. Rows represent predicted classes and columns represent reference classes. The background row contains 35 missed reference regions, and the background column contains 17 unmatched predictions.

The confusion matrix, which analyzes the model’s performance class-by-class, is shown in Figure 9.

In this study, high values along the diagonal of the matrix indicate that the model often correctly classifies. Most misclassifications occurred between adjacent CVM stages.

On the 344-image test set, the model correctly detected and classified 277 of 344 reference CVM regions (80.52%). There were 32 incorrect stage assignments and 35 missed reference regions. The model produced 326 predictions, including 17 unmatched predictions, yielding a micro-averaged precision of 84.97% (277/326). Micro-averaged recall and F1-score were 80.52% and 82.69%, respectively. Class-specific results are presented in Table 6.

### 3.3. Results on the Performance of the Combined Diagnostic Model

Demographic characteristics and reference CVM and MPS stage distributions of the training subset (*n* = 260) and held-out test subset (*n* = 65) are presented in Supplementary Table S4.

Comparative performance metrics across all baseline configurations and the combined model on the held-out internal test set (*n* = 65) are presented in Table 7.

The age-only model achieved a five-class accuracy of 41.5% and a macro-F1 score of 0.428. Models using AI-derived and manually assessed CVM stages alone achieved accuracies of 56.9% and 60.0%, respectively. The combined model incorporating AI-derived CVM stage, chronological age, and sex achieved an accuracy of 69.2% (45/65; 95% CI: 57.2–79.1%), a macro-F1 score of 0.704, a linearly weighted kappa of 0.724, and a quadratically weighted kappa of 0.853. Within-one-stage agreement was 98.5% (64/65). The same fitted model evaluated with reference CVM stage, age, and sex achieved an accuracy of 67.7%.

Additional internal validation used nested, patient-grouped five-fold cross-validation with five repeats, yielding 25 outer-fold evaluations. Across the development cohort, the model incorporating manual CVM, chronological age, and sex achieved a mean five-class accuracy of 58.27% (95% CI: 53.09–63.23%) and a mean macro-F1 score of 0.583. The age-only model achieved 44.73% accuracy (95% CI: 40.14–49.30%) and a macro-F1 score of 0.384. Manual CVM alone achieved 58.80% accuracy and a macro-F1 score of 0.591, whereas age and sex together achieved 51.87% accuracy and a macro-F1 score of 0.440. Among the 65 patients with available AI-CVM outputs, substituting AI-CVM during outer-fold evaluation without refitting the reference-CVM models yielded a mean accuracy of 55.69% (95% CI: 45.23–66.46%) and a macro-F1 score of 0.590 (Table 8).

Confusion matrices comparing AI-predicted CVM stages and combined mod-el-predicted MPS stages with their respective reference stages in the 65 held-out patients are presented in Table 9 and Table 10.

YOLOv11 returned at least one detection for all 65 held-out test cephalograms; when more than one box was returned, the highest-confidence prediction was used (Supplementary Figure S1). No patient was excluded.

When MPS stages were grouped as A–C versus D–E, the model correctly classified 60 of 65 patients (92.3%; 95% CI: 83.2–96.7%). With A–C designated as the positive category, sensitivity was 85.0% (17/20; 95% CI: 64.0–94.8%) and specificity was 95.6% (43/45; 95% CI: 85.2–98.8%). Positive and negative predictive values were 89.5% (17/19; 95% CI: 68.6–97.1%) and 93.5% (43/46; 95% CI: 82.5–97.8%), respectively. Three of the ten reference stage-C cases were classified as D; no reference stage-C cases were classified as E. One reference stage-D case and one reference stage-E case were classified as C. Separately, within-one-stage agreement was 98.5% (64/65; 95% CI: 91.8–99.7%); the only prediction differing from the reference by more than one stage was the stage-E case classified as C.

## 4. Discussion

This research aims to develop and evaluate the effectiveness of an AI-assisted automated classification system for assessing skeletal growth and midpalatal suture maturation, which are critically important for orthodontic diagnosis and treatment planning.

In previous studies examining the relationship between CVM and MPS, which constitutes the first stage of the research, high correlation values (r = 0.858 and r = 0.908) were reported by Hezenci and Bulut [11] and Angelieri et al. [12]; it was emphasized that CS1–CS3 stages are reliable indicators for early MPS stages, and that individual CBCT assessment is necessary in the postpubertal period. In another similar study, RME was recommended for CVM stages CS1–CS3, but not for patients with CVM stages CS4–CS5 [25]. In this study, data from 250 individuals were analyzed, and a significant and strong positive correlation was found (r = 0.749; *p* < 0.001). In the multivariable categorical CVM model, chronological age remained positively associated with MPS stage, while lower CVM stages were associated with lower cumulative odds of more advanced MPS maturation relative to CS6. Nevertheless, the early-stage cross-tabulations were sparse, and the very small CS1–CS3 odds ratios should not be treated as precise clinical effect sizes. Modeling CVM as a single ordinal score or combining CS1–CS3 reduced model fit; the single-score model also showed evidence against proportional odds. These sensitivity analyses support retaining categorical CVM for the present analysis while acknowledging the uncertainty of early-stage estimates. The overlap of MPS stages, particularly at CS4 and CS5, further limits inference about an individual patient’s sutural maturation from CVM alone.

Sex-related patterns were also examined. Previous studies have reported that females tend to exhibit more advanced MPS maturation than males, although findings regarding the association between sex and MPS stage have varied [6,7,8,26]. In the present study, the correlation between CVM and MPS stage was numerically stronger in males (r = 0.851; *p* < 0.001) than in females (r = 0.680; *p* < 0.001). However, sex was not significantly associated with MPS stage in the multivariable model incorporating CVM stage and chronological age (male vs. female: adjusted OR = 0.673; 95% CI: 0.364–1.244; *p* = 0.206). The unadjusted CVM–MPS correlation was higher in males than in females, with a bootstrap confidence interval for the difference excluding zero. However, females were more concentrated in CS5–CS6 (76.4% vs. 61.8%), and the comparison did not adjust for these different stage distributions. Restricted range and unequal distributions may contribute to the observed correlation difference. The observed correlation difference therefore does not establish sex-specific biological maturation patterns. Whether sex modifies the CVM–MPS relationship requires further investigation accounting for differences in age and maturation-stage distributions.

The second phase of the research, AI-assisted automated CVM classification, has gained significant traction in the literature as AI applications have revolutionized dentistry in recent years. Recent advancements in convolutional neural network (CNN) architectures using deep learning have enabled the development of direct inference, recognition, judgment, and classification capabilities [27]. With these advancements, CNN architectures are widely used in dentistry to perform tasks such as detecting anatomical structures or pathologies, segmenting them and classifying them [28]. CNN architectures have also been frequently used in automated CVM classification, occupying a large place in the literature, and were the preferred architecture in this research. The CNN model developed by Akay et al. [17] took values in the range of 0.47–0.82 Accuracy; 0.37–0.74 Recall; 0.44–0.76 F1-Score for each CVM stage and achieved an average success rate of 58.66% accuracy. The DetNet model used in a similar study achieved an overall accuracy of 71% for CVM staging [18]. In a study comparing the performance of different CNN architectures (VGG16, GoogLeNet, DenseNet161, and ResNet152) in automatic CVM classification, ResNet152 achieved the highest accuracy of 67.06% [19].

With the ongoing development of deep learning methods, research has focused on improving model performance by exploring feasible alternatives. In this context, the real-time performance and flexibility in a wide range of research areas have solidified You Only Look Once (YOLO) as a leading algorithm in both academic research and practical applications [29]. It is used for a very wide range of medical diagnoses, such as mandibular fracture, rapid brain tumor detection on magnetic resonance imaging, coronary artery segmentation, and stenosis detection [30,31,32]. Segmentation of the cervical vertebral body from X-ray and magnetic resonance images is another application area of YOLO architectures [33]. However, a literature review revealed only a few studies on automatic CVM classification using YOLO architectures. The YOLOv3-based model developed by Li et al. [20] achieved good performance for CVM evaluation with a total accuracy of 70.42%. Jiang et al. [21] developed a target-detection model, DC-YOLOv5, for fully automatic detection of the CVM stage and compared it with YOLOv3, YOLOv4, YOLOv5, and YOLOv7. DC-YOLOv5 was reported to achieve superior performance compared to other algorithms, with a general F1 score of 0.993. A literature search did not reveal any research on automatic CVM classification using YOLOv11. A similar study by Kayaoğlu et al. [34] showed that the YOLOv8 and YOLOv11 models, used for the detection and semantic segmentation of C2, C3, and C4 cervical vertebrae without automatic CVM classification, achieved 99.8% accuracy, enabling rapid and effective detection of cervical vertebral regions.

In this study, YOLOv11 correctly detected and classified 80.5% of reference CVM regions when missed detections were included in the denominator. This finding suggests potential for automated CVM assessment, while highlighting the need to consider both localization failures and incorrect stage assignments when evaluating performance. The higher micro-averaged precision (85.0%) reflects the proportion of predictions that were correct and does not account for missed reference regions. Comparisons with previously reported classification accuracies should therefore be interpreted cautiously, given differences in metric definitions, localization requirements, datasets, and evaluation protocols.

The third and final stage of this research, the development of a combined diagnostic model with artificial intelligence, is becoming increasingly important to overcome the limitations of methods based on a single modality. Indeed, Huang et al. [35] reported that systems combining medical images and electronic health records with deep learning-based fusion methods performed better than models based solely on imaging data. The most notable examples of combined models are the simultaneous use of stimulated Raman histology and CNN-based deep learning systems in intraoperative brain tumor diagnosis, and the pathology baseline model, which achieves strong success in cancer diagnosis and prognosis prediction by integrating image data with clinical information [36,37].

Baseline comparisons provided context for interpreting the combined model’s performance. The heuristic rule grouping CS1–CS3 as MPS stages A–C and CS4–CS6 as stages D–E achieved binary accuracies of 85.6% in Dataset 1 and 89.2% (58/65) in the held-out test set. In the same test set, the combined model achieved 92.3% accuracy (60/65), corresponding to only two additional correct predictions and an absolute difference of approximately 3.1 percentage points. This small numerical difference does not establish superiority over the heuristic rule. The combined model also predicted individual MPS stages and achieved 98.5% within-one-stage agreement. However, this measure does not distinguish errors that cross the C–D boundary from those occurring within the A–C or D–E categories. Models using AI-derived and manually assessed CVM stages alone achieved accuracies of 56.9% and 60.0%, respectively; the corresponding models incorporating age and sex achieved 69.2% and 67.7%. The small numerical difference between the two CVM input configurations does not establish superiority or equivalence. These comparisons also do not isolate the contributions of CVM classification errors, biological variability, reference-label uncertainty, and model limitations to prediction error. Although the combined models achieved numerically higher accuracies than the corresponding CVM-only models in the held-out test set, adding age and sex did not improve mean cross-validation accuracy over manual CVM alone (58.27% versus 58.80%). Thus, the cross-validation results did not demonstrate an incremental benefit of age and sex in terms of mean five-class accuracy.

All five binary misclassifications crossed the C–D threshold used to distinguish stages A–C from D–E: three reference stage-C cases were predicted as D, and one reference stage-D and one stage-E case were predicted as C. Thus, the high binary accuracy (92.3%) does not eliminate uncertainty at this threshold. These errors demonstrate that the model may both overestimate and underestimate MPS maturation, limiting its use as a standalone basis for expansion planning. The reported performance reflects agreement with CBCT-based maturation categories; expansion outcomes were not evaluated. Therefore, these findings do not establish the model’s ability to predict expansion success or justify replacing clinically indicated CBCT examinations. External validation is required before clinical implementation.

Recent studies have demonstrated several approaches to AI-assisted MPS assessment. Milani et al. [22] developed an end-to-end framework for classifying MPS maturation stages from CBCT images. Cai et al. [23] introduced a multimodal model combining CBCT and cephalometric images with clinical variables, including age, sex, CVM stage, and third molar development. Lu et al. [24] directly predicted CBCT-defined MPS maturity from lateral cephalograms, reporting an external-test accuracy of 0.93 and Cohen’s kappa of 0.86. Their prediction task was binary, distinguishing immature from mature sutures, whereas the present model predicts five MPS stages, with an additional secondary evaluation after grouping predictions into A–C and D–E. Differences in classification targets, populations, reference standards, and validation designs preclude direct comparisons of performance.

The contribution of the present study lies in its two-stage structure: automated CVM classification provides an explicit intermediate assessment, which is subsequently combined with chronological age and sex to predict MPS stage. This approach requires no CBCT input at the prediction stage, although CBCT-derived labels were required for model development and evaluation. The intermediate CVM output allows skeletal staging and subsequent MPS prediction to be examined separately; however, CVM classification errors may propagate to the second stage, and potentially relevant cephalometric information beyond CVM is not directly incorporated into the MPS predictor. The model should therefore be regarded as a complementary approach requiring external validation.

This study has several limitations. First, its single-center design may limit generalizability to other populations and imaging devices. The small held-out test set (*n* = 65) also limits the precision of performance estimates. In particular, the test set contained only three stage-A and seven stage-B cases, limiting the precision of stage-specific performance estimates. The female predominance and concentration of CS6 cases in Dataset 1 may also limit the generalizability of the observed CVM–MPS associations to populations with different sex and maturation-stage distributions. Several methodological limitations concern the combined-model pipeline. The model was fitted using reference CVM stages and applied using automated stages; consequently, errors arising from automated CVM assessment were not represented during model fitting. Automated outputs were generated only for the 65 held-out test patients, all of whom received a CVM prediction and were retained in the evaluation. Thus, evaluation with automated inputs was restricted to this subset. The cross-validation estimate with AI inputs in this subset was 55.7% (95% CI: 45.2–66.5%), with the wide confidence interval indicating substantial uncertainty in the performance estimate. Consequently, the complete automated pipeline has not been evaluated across the full cohort, and the reference-input cross-validation results should not be interpreted as validation of the complete automated pipeline. The held-out model used hyperparameters selected in a preliminary analysis that excluded all 65 final test patients, whereas repeated nested cross-validation re-selected hyperparameters within each outer training partition. The resulting accuracies of 69.2% in the held-out test set, 58.27% with reference CVM across all 325 patients, and 55.69% after AI-CVM substitution during evaluation in the 65-patient subset therefore represent different evaluation settings and should not be interpreted as interchangeable estimates (Table 8). Probability calibration and paired uncertainty estimates for comparisons with baseline models were not assessed, which restricts assessment of probability reliability and comparative performance. Second, classification errors occurred most frequently between adjacent stages, potentially reflecting gradual biological transitions and the difficulty of classifying borderline cases. Moreover, the limited reproducibility of the Angelieri classification reported by Barbosa et al. [38] may introduce uncertainty into the reference labels; agreement with these labels does not establish biological accuracy. Further optimization of CNN architectures may improve classification performance, particularly at stage boundaries. Additional determinants, including vertical skeletal pattern, tooth mineralization, sutural density, and alternative MPS classifications, were not evaluated. Multicenter studies with external validation and broader clinical variables are needed to assess clinical applicability.

## 5. Conclusions

This study demonstrated a strong positive correlation between CVM and MPS stages. In the multivariable ordinal logistic regression model, CVM stage and chronological age were significantly associated with MPS stage, whereas sex was not. An AI-assisted model integrating automated CVM classification, chronological age, and sex was also developed. YOLOv11 correctly detected and classified 277 of 344 reference CVM regions (80.5%), with micro-averaged precision, recall, and F1-score of 85.0%, 80.5%, and 82.7%, respectively. The combined model, trained with reference CVM, age, and sex and applied using AI-predicted CVM, achieved 69.2% accuracy in the 65-patient held-out test set. Repeated nested cross-validation across 325 patients yielded 58.3% accuracy with reference CVM and 55.7% when AI-CVM was substituted only during evaluation in the 65-patient subset. The combined model remains an investigational approach for estimating CBCT-defined MPS maturation categories, with evaluation of the complete automated pipeline limited to 65 patients. The added predictive value of age and sex beyond CVM staging alone remains unproven. Agreement with these categories does not establish the ability to predict expansion success, and the model should not replace clinically indicated CBCT examinations. External validation in larger, multicenter cohorts representative of the intended clinical population is required before clinical implementation.

## Figures and Tables

**Table 1:** Summary of datasets used in the study.

Dataset	Purpose	Sample Size (*n*)	Data Included	Source/Determination Method	Application
Dataset 1	Correlation Analysis	250 (89M, 161F)	CVM (manual), MPS (manual), Chronological Age, Sex	Akdeniz University Orthodontic Archive (2019–2024)	Statistical analysis of CVM-MPS relationship
Dataset 2	YOLOv11 Model	1448	Cephalometric Radiographs, CVM (manual)	Akdeniz University Radiology Archive	Training the AI model for automated CVM classification
Dataset 3	Combined Model	325	Reference CVM and MPS stages, Chronological Age, Sex	Akdeniz University Orthodontic Archive (2019–2025)	Model fitting with reference CVM; evaluation with reference or AI-CVM inputs

Dataset 3 comprised all 250 patients from Dataset 1 and 75 additional patients and was divided into training (*n* = 260) and test (*n* = 65) subsets. Dataset 2 had no patient-level overlap with either Dataset 1 or Dataset 3.

**Table 2:** Demographic and maturation stage distribution of the study population used for correlation analysis (Dataset 1, *n* = 250).

Variable	Category	Frequency (*n*)	Percentage (%)
Sex	Male	89	35.6
	Female	161	64.4
CVM Stage	CS1	14	5.6
	CS2	18	7.2
	CS3	20	8.0
	CS4	20	8.0
	CS5	64	25.6
	CS6	114	45.6
MPS Stage	A	9	3.6
	B	27	10.8
	C	50	20.0
	D	76	30.4
	E	88	35.2

**Table 3:** Distribution of MPS stages across cervical stages and correlation analysis (Dataset 1, *n* = 250).

		MPS					Total (*n*)	R (Spearman’s Rho)	95% CI for R
		A	B	C	D	E			
**CVM Stage**	**CS1**	9	4	1	-	-	14	0.749 (<0.001)	0.675–0.811
	**CS2**	-	15	3	-	-	18		
	**CS3**	-	7	12	1	-	20		
	**CS4**	-	1	8	11	-	20		
	**CS5**	-	-	18	30	16	64		
	**CS6**	-	-	8	34	72	114		
Total (*n*)		9	27	50	76	88	250		

R, Spearman rank correlation; CI, confidence interval. Two-sided *p*-values were obtained using 99,999 random permutations. Percentile confidence intervals were based on 50,000 participant-level bootstrap resamples.

**Table 4:** Distribution and correlation analysis of midpalatal suture maturation stages according to sex and CVM stage (Dataset 1, *n* = 250).

		MPS					R (Spearman’s Rho)	95% CI for R
Sex	CVM Stage	A	B	C	D	E		
M	**CS1**	4	1	1	-	-	0.851 (<0.001)	0.769–0.907
	**CS2**	-	12	2	-	-		
	**CS3**	-	2	7	1	-		
	**CS4**	-	-	-	4	-		
	**CS5**	-	-	4	12	2		
	**CS6**	-	-	1	14	22		
F	**CS1**	5	3	-	-	-	0.680 (<0.001)	0.571–0.772
	**CS2**	-	3	1	-	-		
	**CS3**	-	5	5	-	-		
	**CS4**	-	1	8	7	-		
	**CS5**	-	-	14	18	14		
	**CS6**	-	-	7	20	50		

R, Spearman rank correlation; CI, confidence interval. Two-sided *p*-values were obtained using 99,999 random permutations. Percentile confidence intervals were based on 50,000 participant-level bootstrap resamples.

**Table 5:** Associations of CVM stage, sex, and chronological age with midpalatal suture maturation stage: multivariable ordinal logistic regression (Dataset 1, *n* = 250).

Category	β	SE	Adjusted OR	95% CI for OR	*p*-Value
CS1	−9.774	1.486	0.000057	0.000003–0.001048	<0.001
CS2	−6.262	1.036	0.001907	0.000250–0.014526	<0.001
CS3	−4.401	0.890	0.012	0.002–0.070	<0.001
CS4	−1.465	0.578	0.231	0.074–0.717	0.011
CS5	−0.993	0.351	0.370	0.186–0.737	0.005
Sex	−0.396	0.314	0.673	0.364–1.244	0.206
Chronological age	0.272	0.065	1.313	1.155–1.491	<0.001

SE, standard error; OR, odds ratio; CI, confidence interval. Reference categories are CS6 for CVM stage and female for sex. The age OR is expressed per year. Wald 95% confidence intervals are reported. Profile-likelihood confidence intervals for CS1–CS3 are provided in Supplementary Table S2, and model thresholds are reported in Supplementary Table S3.

**Table 6:** Class-specific CVM detection and staging performance in the test subset of Dataset 2 (*n* = 344).

CVM Stage	Reference Regions (*n*)	Precision (%)	Recall (%)	F1-Score (%)
CS1	54	86.79	85.19	85.98
CS2	63	81.97	79.37	80.65
CS3	65	86.89	81.54	84.13
CS4	56	82.69	76.79	79.63
CS5	53	83.33	75.47	79.21
CS6	53	88.24	84.91	86.54
Macro average	—	84.98	80.54	82.69
Micro average	344	84.97	80.52	82.69

Precision includes unmatched predictions, whereas recall includes missed reference regions. Macro averages give equal weight to the six CVM classes; micro averages pool counts across classes. Background is not treated as a separate CVM stage.

**Table 7:** Performance comparison of baseline predictors, the heuristic rule, and combined diagnostic models on the held-out test set (*n* = 65).

Model/Predictor	Inputs	5-Class Accuracy	Macro-F1	Weighted-F1	Linear Weighted κ	Quadratic Weighted κ	Binary Accuracy (A–C vs. D–E)	Within-1-Stage Agreement
Heuristic Clinical Rule	CS1–3 (Open), CS4–6 (Fused)	N/A *	N/A	N/A	N/A	N/A	0.892 (58/65; 0.794–0.947)	N/A
Baseline 1: Age Alone	Chronological Age	0.415 (27/65; 0.304–0.537)	0.428	0.393	0.447	0.669	0.815 (53/65; 0.704–0.891)	0.938 (61/65)
Baseline 2: AI-CVM Alone	YOLOv11 CVM Stage	0.569 (37/65; 0.448–0.682)	0.493	0.540	0.620	0.792	0.908 (59/65; 0.813–0.957)	0.969 (63/65)
Baseline 3: Manual CVM Alone	Manual CVM Stage	0.600 (39/65; 0.479–0.710)	0.518	0.578	0.653	0.819	0.923 (60/65; 0.832–0.967)	0.985 (64/65)
Manual-CVM reference model	Manual CVM + Age + Sex	0.677 (44/65; 0.556–0.778)	0.691	0.677	0.714	0.851	0.923 (60/65; 0.832–0.967)	0.985 (64/65)
Proposed Combined Model	AI-CVM + Age + Sex	0.692 (45/65; 0.572–0.791)	0.704	0.693	0.724	0.853	0.923 (60/65; 0.832–0.967)	0.985 (64/65)

Five-class and binary accuracies are reported as proportions (correct predictions/total; 95% Wilson confidence interval). Within-one-stage agreement is the proportion of predictions within one stage of the reference (*n*/total). Confidence intervals are model-specific and do not represent paired comparisons. * N/A: not applicable; the heuristic produces binary categories only.

**Table 8:** Internal validation estimates of the combined model, by evaluated population, model fitting and CVM input.

Analysis	Patients Evaluated	Fitted Models	CVM at Model Fitting	CVM at Evaluation	5-Class Accuracy, % (95% CI)	Macro-F1
Single held-out split	65 (test subset)	1 (fitted on 260)	Reference (manual)	YOLOv11	69.2 (57.2–79.1)	0.704
Single held-out split	65 (test subset)	Same model	Reference (manual)	Reference (manual)	67.7 (55.6–77.8)	0.691
Repeated nested cross-validation	325 (all)	25 (5 folds × 5 repeats)	Reference (manual)	Reference (manual)	58.27 (53.09–63.23)	0.583
Repeated nested cross-validation, AI substitution	65 (AI outputs available)	Same 25 models	Reference (manual)	YOLOv11	55.69 (45.23–66.46)	0.590

In the AI-substitution analysis, each patient contributed predictions only from outer-fold models for which that patient was excluded from training. AI-CVM was substituted only at evaluation; the fitted models were not updated.

**Table 9:** Confusion matrix comparing manually determined and AI-predicted CVM stages in the held-out internal test set (Dataset 3, *n* = 65).

Manual/AI-Predicted	CS1	CS2	CS3	CS4	CS5	CS6	Total
CS1	4	1	0	0	0	0	5
CS2	1	4	0	0	0	0	5
CS3	0	0	4	1	0	0	5
CS4	0	0	0	5	0	0	5
CS5	0	0	0	0	15	3	18
CS6	0	0	0	1	0	26	27
Total	5	5	4	7	15	29	65

Linearly weighted κ = 0.926 (95% CI: 0.861–0.974); quadratically weighted κ = 0.971 (95% CI: 0.933–0.992).

**Table 10:** Confusion matrix comparing manually determined and combined model-predicted MPS stages in the held-out internal test set (Dataset 3, *n* = 65).

Manual/Model-Predicted	MPS A	MPS B	MPS C	MPS D	MPS E	Total
MPS A	2	1	0	0	0	3
MPS B	0	5	2	0	0	7
MPS C	0	1	6	3	0	10
MPS D	0	0	1	18	5	24
MPS E	0	0	1	6	14	21
Total	2	7	10	27	19	65

Linearly weighted κ = 0.724 (95% CI: 0.587–0.829); quadratically weighted κ = 0.853 (95% CI: 0.743–0.919).

## Data Availability

Data underlying this article will be shared upon reasonable request to the corresponding author. Individual-level data are anonymized and subject to institutional restrictions.

## Supplementary Materials

The following supporting information can be downloaded at: https://www.mdpi.com/article/10.3390/diagnostics16213568/s1. Table S1. Model fit under alternative CVM representations; Table S2. Profile likelihood confidence intervals for early CVM stages; Table S3. Sensitivity model coefficients and thresholds for all models; Table S4. Demographic characteristics and reference CVM and MPS stage distributions of the training and held-out test subsets of the combined-model cohort (*n* = 325). Figure S1. Example of multiple YOLOv11 detections on a lateral cephalometric radiograph. The interface reports two detected objects; the displayed CVM label is CS3 with a confidence score of 55%. When multiple predictions were returned, the prediction with the highest confidence was retained for the combined-model input. CVM, cervical vertebral maturation.

## Abbreviations

The following abbreviations are used in this manuscript:

CVM	Cervical Vertebral Maturation
MPS	Midpalatal Suture Maturation
AI	Artificial Intelligence
CBCT	Cone-Beam Computed Tomography
wk	Weighted Kappa
RME	Rapid Maxillary Expansion
CS	Cervical Stage
JPEG	Joint Photographic Experts Group
DICOM	Digital Imaging and Communications in Medicine
CNN	Convolutional Neural Network
YOLO	You Only Look Once
