# Supplementary material for: Artificial Intelligence-Assisted Prediction of Midpalatal Suture Maturation from Cervical Vertebral Maturation Assessed on Lateral Cephalometric Radiographs: Development of a Combined Diagnostic Model
Source: Diagnostics (Basel). 2026 Oct 29;16(21):3568. doi: 10.3390/diagnostics16213568 (PMC13762103; doi:10.3390/diagnostics16213568)
Supplement: Supplementary file 1 [file diagnostics-16-03568-s001.zip › Supplementary_Figure_S1.pdf]

Supplementary Figure S1

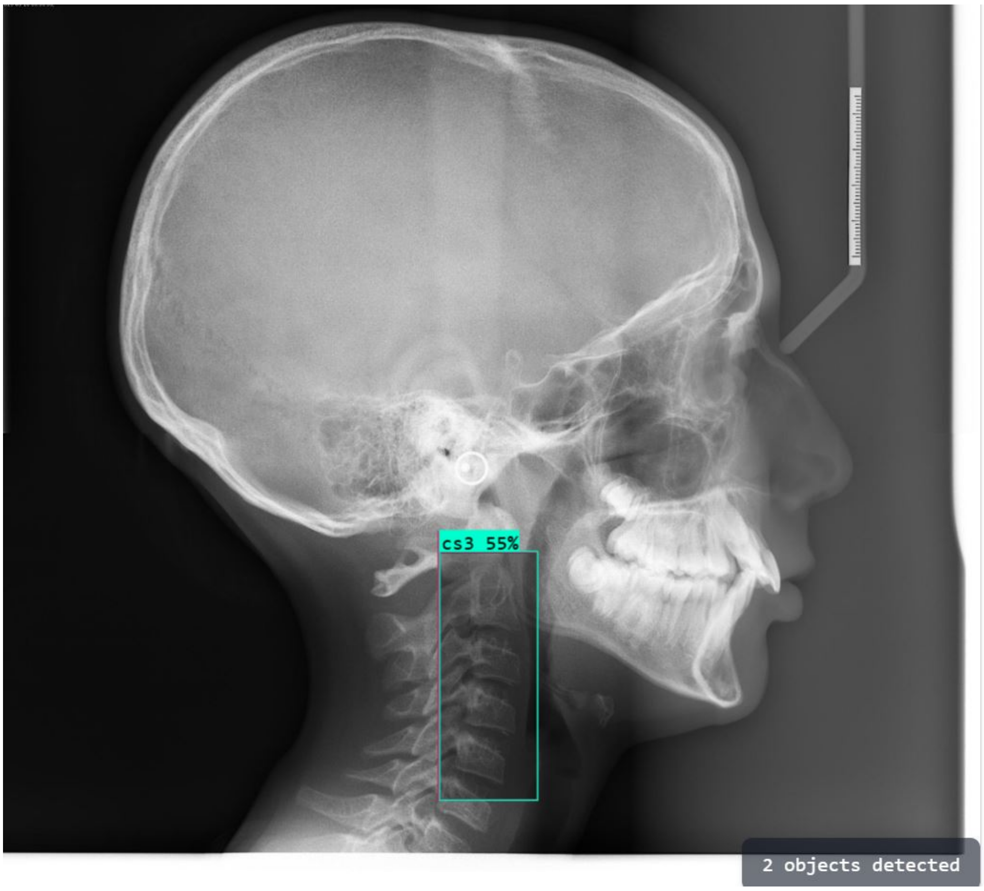

Figure S1. Example of multiple YOLOv11 detections on a lateral cephalometric radiograph. The interface reports two detected objects; the displayed CVM label is CS3 with a confidence score of 55%. When multiple predictions were returned, the prediction with the highest confidence was retained for the combined-model input. CVM, cervical vertebral maturation.
