# Supplementary material for: Artificial Intelligence-Assisted Prediction of Midpalatal Suture Maturation from Cervical Vertebral Maturation Assessed on Lateral Cephalometric Radiographs: Development of a Combined Diagnostic Model
Source: Diagnostics (Basel). 2026 Oct 29;16(21):3568. doi: 10.3390/diagnostics16213568 (PMC13762103; doi:10.3390/diagnostics16213568)
Supplement: Supplementary file 1 [file diagnostics-16-03568-s001.zip › Supplementary_Tables.pdf]

Supplementary Tables

Table S1. Model fit under alternative CVM representations

CVM representation	Parameters[1]	Log likelihood	AIC	LR test vs categorical[2]	PO score p
Categorical (primary)	11	−212.953	447.907	Reference	0.065
Single score (1–6)	7	−220.133	454.267	χ^2=14.360; df=4; p=0.006	0.013
CS1–CS3 combined	9	−228.487	474.975	χ^2=31.068; df=2; p<0.001	0.123

All models included the same 250 participants, MPS stages ordered A–E, chronological age, and sex (female reference). The primary model treated CVM as six categories (CS6 reference). The grouped model used CS1–CS3, CS4, CS5, and CS6 (reference). Sensitivity analyses assessed whether simpler CVM representations could address concerns about sparse early-stage estimates.

[1]Number of estimated parameters, including four thresholds. [2]Likelihood-ratio tests compare each restricted model with the primary categorical model. Their chi-square calibration is asymptotic and may be affected by sparsity. AIC, Akaike information criterion; LR, likelihood ratio; PO, proportional odds. Lower AIC indicates better relative fit. A nonsignificant PO test does not establish that the assumption holds.

Table S2. Profile likelihood confidence intervals for early CVM stages

Contrast	β	Adjusted OR	Profile likelihood 95% CI for OR
CS1 vs CS6	-9.774	5.691×10^{-5}	1.761×10^{-6} to 7.610×10^{-4}
CS2 vs CS6	-6.262	1.907×10^{-3}	1.951×10^{-4} to 1.247×10^{-2}
CS3 vs CS6	-4.401	1.227×10^{-2}	1.574×10^{-3} to 6.183×10^{-2}

Estimates are from the primary categorical CVM model, including chronological age and sex. Confidence intervals were obtained by profiling each coefficient while reoptimizing all remaining slopes and thresholds, using the $\chi^2(1)$=3.841 likelihood-ratio cutoff. These intervals complement the Wald intervals in Table 5. All estimates and intervals were finite, but their broad ranges do not eliminate concerns about sparse-data bias or imprecision. OR, odds ratio; CI, confidence interval.

Numerical checks: five dispersed starting values converged to the same log-likelihood (−212.953491), with maximum parameter differences below 2.4×10^{-8} and a positive-definite observed Hessian. Finite, reproducible estimates should not be interpreted as proof that sparse-data bias is absent.

Table S3. Sensitivity model coefficients and thresholds for all models

A Coefficients for the sensitivity models

Model	Predictor	β	SE	OR	95% CI for OR	p
Single score	CVM, per stage	1.487	0.185	4.424	3.076–6.363	<0.001
Single score	Male vs female	-0.372	0.306	0.689	0.378–1.257	0.225
Single score	Age, per year	0.219	0.062	1.245	1.102–1.407	<0.001
CS1–CS3 combined	CS1–CS3 vs CS6	-5.318	0.882	0.005	8.705×10^{-4} to 2.763×10^{-2}	<0.001
CS1–CS3 combined	CS4 vs CS6	-1.325	0.576	0.266	0.086–0.822	0.021
CS1–CS3 combined	CS5 vs CS6	-0.926	0.350	0.396	0.199–0.786	0.008
CS1–CS3 combined	Male vs female	-0.427	0.305	0.653	0.359–1.187	0.162
CS1–CS3 combined	Age, per year	0.303	0.066	1.354	1.189–1.541	<0.001

All models include age and sex. Wald standard errors and confidence intervals use expected information. The single-score model showed evidence against proportional odds (Table S1); its common OR is a constrained summary. The primary model's predictor coefficients remain in Table 5.

B Thresholds for the primary and sensitivity models

Model	Threshold	Estimate (α_j)	SE
Categorical (primary)	A ∣ B–E	-10.736	1.338
Categorical (primary)	A–B ∣ C–E	-6.155	0.771
Categorical (primary)	A–C ∣ D–E	-2.289	0.308
Categorical (primary)	A–D ∣ E	0.024	0.240
Single score	A ∣ B–E	-1.520	0.596
Single score	A–B ∣ C–E	1.508	0.634
Single score	A–C ∣ D–E	4.700	0.795
Single score	A–D ∣ E	7.179	0.855
CS1–CS3 combined	A ∣ B–E	-8.328	0.856
CS1–CS3 combined	A–B ∣ C–E	-5.939	0.767
CS1–CS3 combined	A–C ∣ D–E	-2.255	0.307
CS1–CS3 combined	A–D ∣ E	0.077	0.239

Parameterization: $\text{logit}[P(\text{MPS} \le j)] = \alpha_j - X\beta$. Age is centered at 16 years. Thresholds for the primary categorical and grouped models refer to a female at CS6 aged 16 years. In the single-score model, CVM is coded as stage minus 1; its thresholds therefore refer to a female at CS1 aged 16 years. Centering changes threshold interpretation, not model fit or slope estimates. Thresholds are on the logit scale and are not clinical age cutoffs; ORs and p-values are not reported for them. SE, standard error; OR, odds ratio; CI, confidence interval.

Table S4. Demographic characteristics and reference CVM and MPS stage distributions of the training and held-out test subsets of the combined-model cohort (n=325).

**Variable**	**Training subset (n=260)**	**Held-out test subset (n=65)**
Female, n (%)	161 (61.9)	43 (66.2)
Male, n (%)	99 (38.1)	22 (33.8)
Age, years, mean ± SD	15.39 ± 3.88	14.97 ± 2.61
CS1, n (%)	21 (8.1)	5 (7.7)
CS2, n (%)	22 (8.5)	5 (7.7)
CS3, n (%)	22 (8.5)	5 (7.7)
CS4, n (%)	20 (7.7)	5 (7.7)
CS5, n (%)	64 (24.6)	18 (27.7)
CS6, n (%)	111 (42.7)	27 (41.5)
MPS A, n (%)	14 (5.4)	3 (4.6)
MPS B, n (%)	32 (12.3)	7 (10.8)
MPS C, n (%)	55 (21.2)	10 (15.4)
MPS D, n (%)	72 (27.7)	24 (36.9)
MPS E, n (%)	87 (33.5)	21 (32.3)

Values are presented as n (%) or mean ± standard deviation. Age is expressed in completed years. CVM and MPS stages were determined by reference assessments.
